# Renal pelvis and ureteropelvic junction incarceration in a Grynfeltt-Lesshaft hernia: a case report and review of the literature

**DOI:** 10.1186/s12894-020-00626-1

**Published:** 2020-06-25

**Authors:** Saadat Mehrabi, Mohammad Javad Yavari Barhaghtalab, Mehdi Babapour

**Affiliations:** Department of General Surgery, Shahid Beheshti Hospital, Yasuj University of Medical Sciences, Yasuj, Iran

**Keywords:** Grynfeltt–Lesshaft hernia, Renal pelvic, Ureteropelvic junction, Incarceration, Hydronephrosis, Muscular or sublay mesh

## Abstract

**Background:**

Grynfeltt–Lesshaft hernia is a kind of lumbar abdominal wall hernia in which clinical presentations may vary from an asymptomatic bulge in the lumbar area to a symptomatic lumbar mass with back pain. It has been accepted to be a rare entity, and incarceration of the kidney through this hernia is shown to be very rare, and very few previous cases have been reported in this regard.

We present a case of renal pelvic and ureteropelvic junction incarceration in a Grynfeltt-Lesshaft hernia and provide an overview of the existing literature on it.

**Case presentation:**

A 76-year-old lady presented to the outpatient clinic with the chief complaint of right flank pain and swelling. Computed tomography (CT) scan of the abdomen was revealed a large herniated sac (60*30 mm) in the upper lumbar triangle with protrusion of retroperitoneal and omental fat, right renal pelvis, ureteropelvic junction and proximal ureter with consecutive hydronephrosis. Herniated retroperitoneal and omental fat was reduced, and closure of the abdominal wall defect was done using retro-muscular Mesh and was fixed to the fascia. The patient was discharged 24 h after the surgery without any complications.

**Conclusion:**

Kidney herniation through the lumbar triangle is extremely rare, and the diagnosis requires careful clinical evaluation. CT scan is the modality of choice for the assessment. Management through surgery should be done in symptomatic patients.

## Background

The lumbar abdominal wall hernia is uncommon. In this kind of hernia retroperitoneal (kidneys, urinary bladder, and ascending or descending colon) and intraperitoneal (small bowel, omentum, preperitoneal fat, stomach, spleen, etc) elements bulge through a defect in the dorsal posterolateral abdominal wall (upper lumbar or the Grynfeltt–Lesshaft triangle) [1–6].

**Fig. 1:**
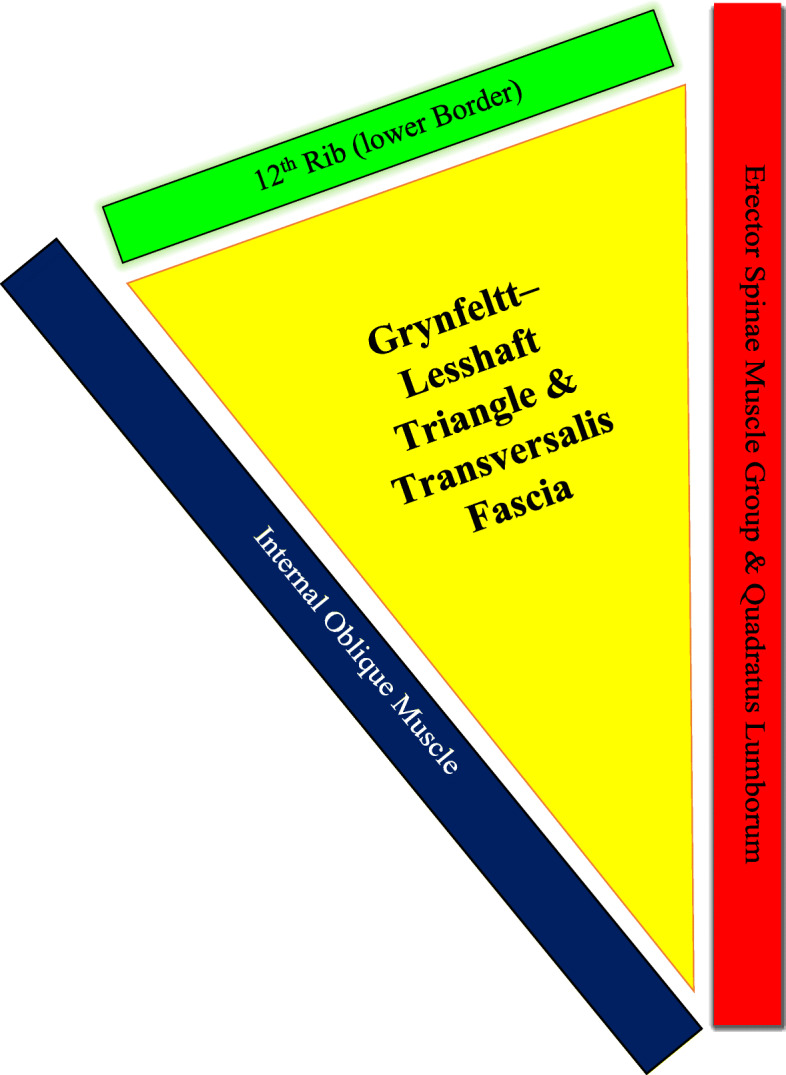
A schematic showing the parameters of the Grynfeltt–Lesshaft Hernia

The Grynfeltt-Lesshaft triangle is defined by the internal oblique muscle laterally, border of the erector spinae muscle group (quadratus lumborum) medially, the 12th rib superiorly, transversalis fascia aponeurosis on the floor, latissimus dorsi muscle on the roof [1, 7, 8]. The weakening of the transversalis fascia and the aponeurosis of the transversus abdominis is the reason for this problem [9] (Fig. 1).

It’s more frequent in men between 50 and 70 years old and is more prevalent on the left side of the abdomen [10]. Risk factors are elderly, obesity, muscular atrophy, and chronic obstructive pulmonary disease (COPD) or any other condition that yields in increasing intra-abdominal pressure [11].

Many surgeons are unfamiliar with lumbar hernias, which may cause a delay in the diagnosis of the disease [3, 7]. Clinical presentations may vary from an asymptomatic bulge in the lumbar area to a symptomatic lumbar mass with back pain or vague abdominal symptoms and are related to the protruded contents [4]. This could have iatrogenic, congenital, and traumatic causes, while most of the patients have primary acquired hernias [1, 4]. Lumbar hernias have been misdiagnosed as lipomas [4, 7, 12–14], muscle strains, fibromas, abscesses, and kidney tumors, causing increased morbidity due to the incorrect diagnosis [3]. It is recommended to repair these hernias electively if there are no contraindications [4]. It has been said that there would be just only one case of lumbar hernia in a general surgeon’s life to operate [13, 15].

A lumbar hernia is an unusual defect, and only 300 cases of primary lumbar hernias have been reported since the first case report by Garangoet in 1731 [13, 14, 16]. Their diagnosis is vital due to their substantial possible complications such as incarceration and strangulation in 25 and 8% of patients retrospectively [17]. So as this kind of hernia has been accepted to be a rare entity, incarceration of the kidney through this hernia is shown to be very rare, and very few previous cases have been reported in this regard [18–21].

We present a case of renal pelvic and ureteropelvic junction herniation in a Grynfeltt-Lesshaft hernia and provide a clear overview of the existing literature on it.

## Case presentation

**Fig. 2:**
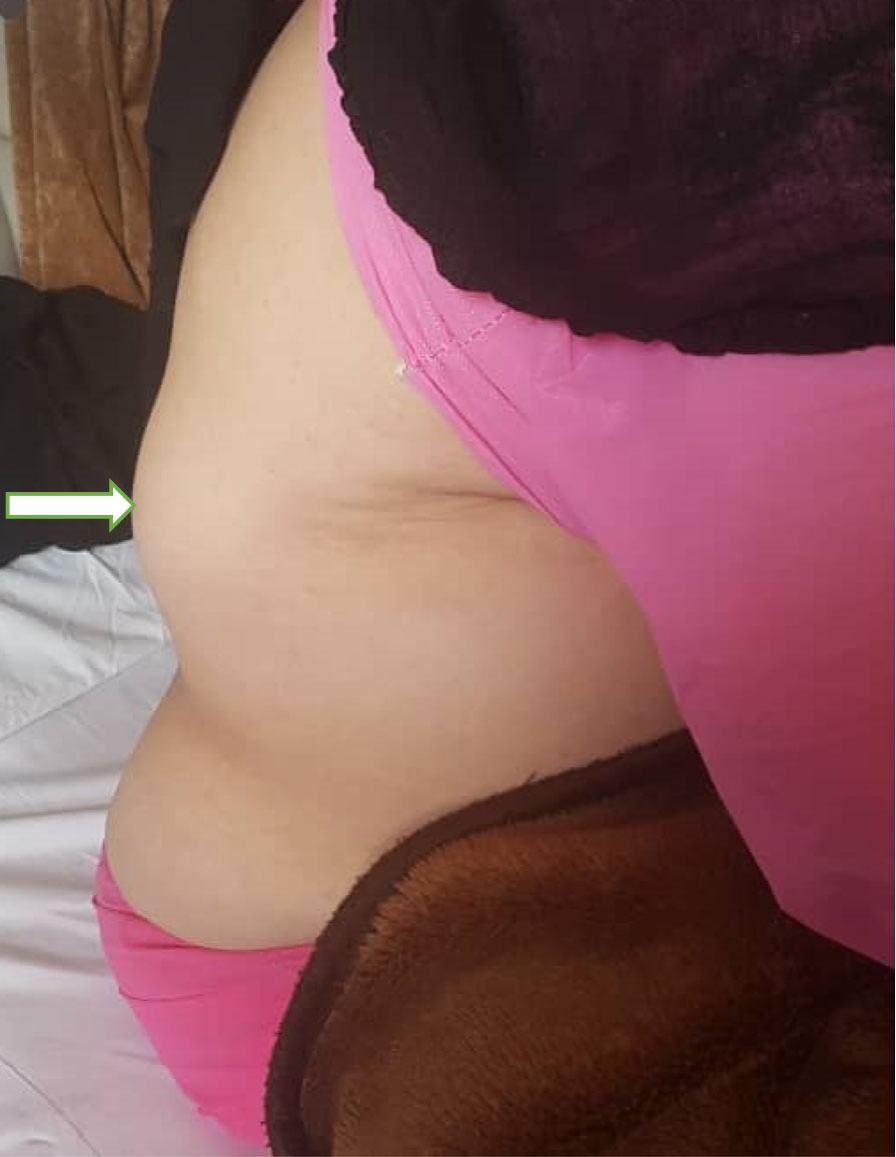
Right lumbar Hernia in a 76-year-old woman is shown with a white arrow

A 76-year-old lady presented to the outpatient clinic with the chief complaint of right flank pain and swelling that protrudes with efforts, reduced mostly in resting position and increasing in size for about 2 months before hospital admission (Fig. 2). The bulging was not associated with gastrointestinal and urinary problems. The patient’s past medical history was positive for diabetes mellitus (DM), hypertension (HTN) and hyperlipidemia (HLP), and an overweight body mass index (BMI) of 28.9. She underwent hysterectomy, laparoscopic cholecystectomy, and urinary bladder prolapse repair surgery previously. She did not have any history of back trauma and heavy lifting previously. She had three children born by vaginal delivery. She used metformin and losartan for medications. There was no family history of the hernia in her parents, siblings, and children. In the physical examination in standing position, we found a soft, mildly tender, irreducible, and smooth border-right lumbar mass (on right scapular line just below the costal margin) which protruded when coughing. No bowel sounds were auscultated on the hernia.

**Fig. 3:**
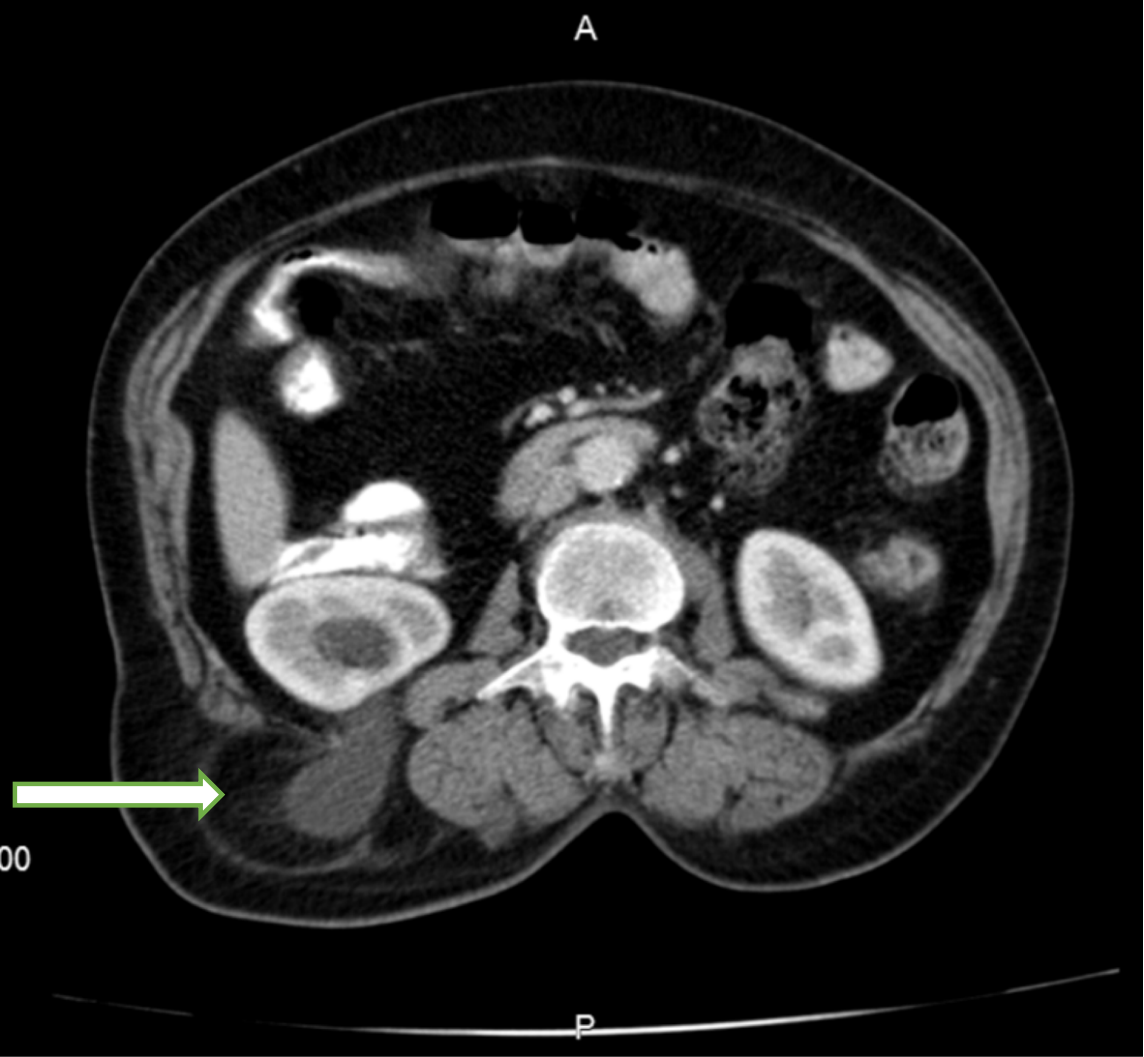
Spiral CT scan without contrast: herniated sac containing retroperitoneal and omental fat, and right renal pelvis (white arrow)

**Fig. 4:**
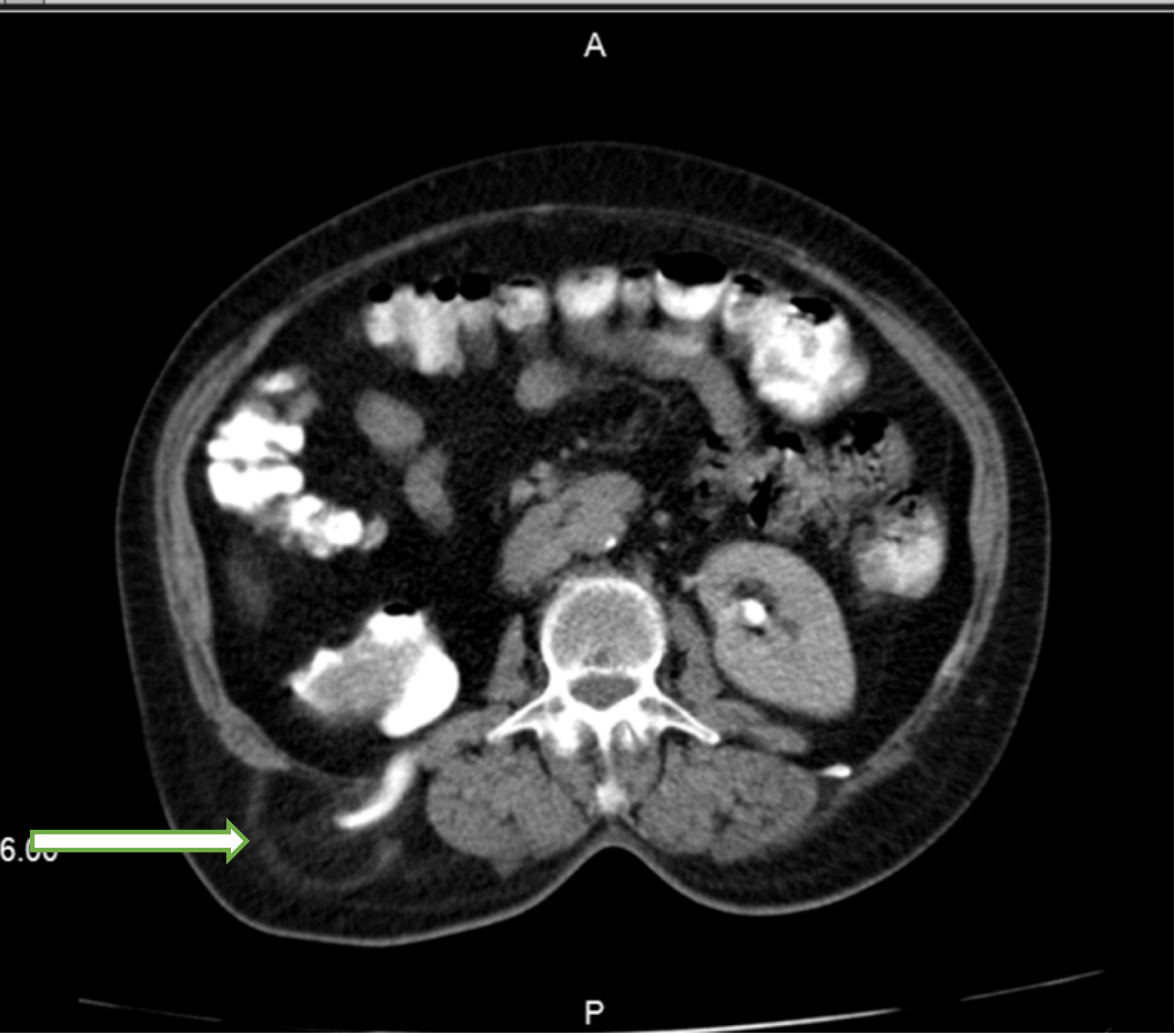
Spiral CT scan with intravenous (IV) contrast: herniated sac containing right ureter (white arrow)

**Fig. 5:**
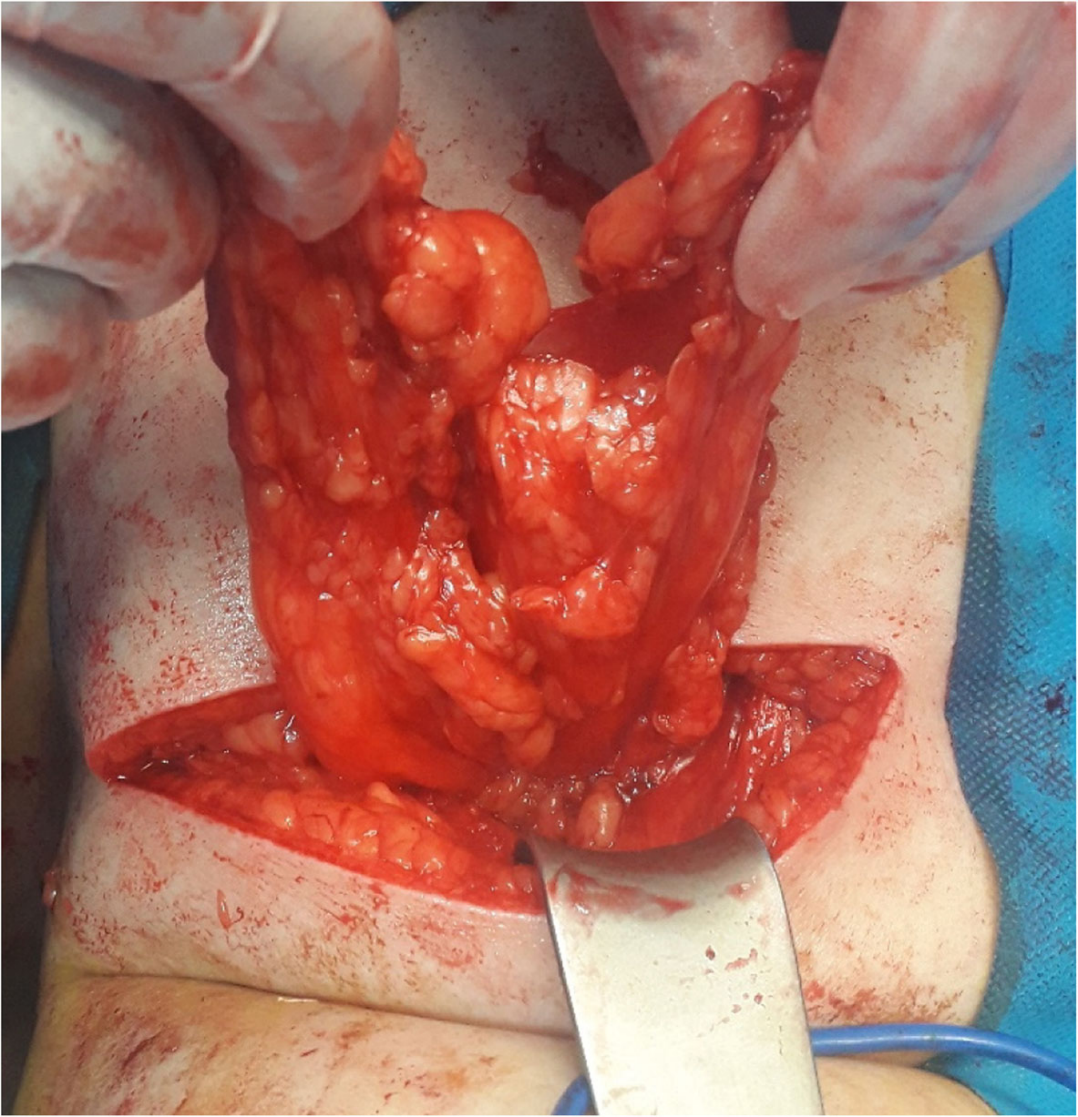
Retroperitoneal and omental fat in the herniated sac in lumbar hernia

**Fig. 6:**
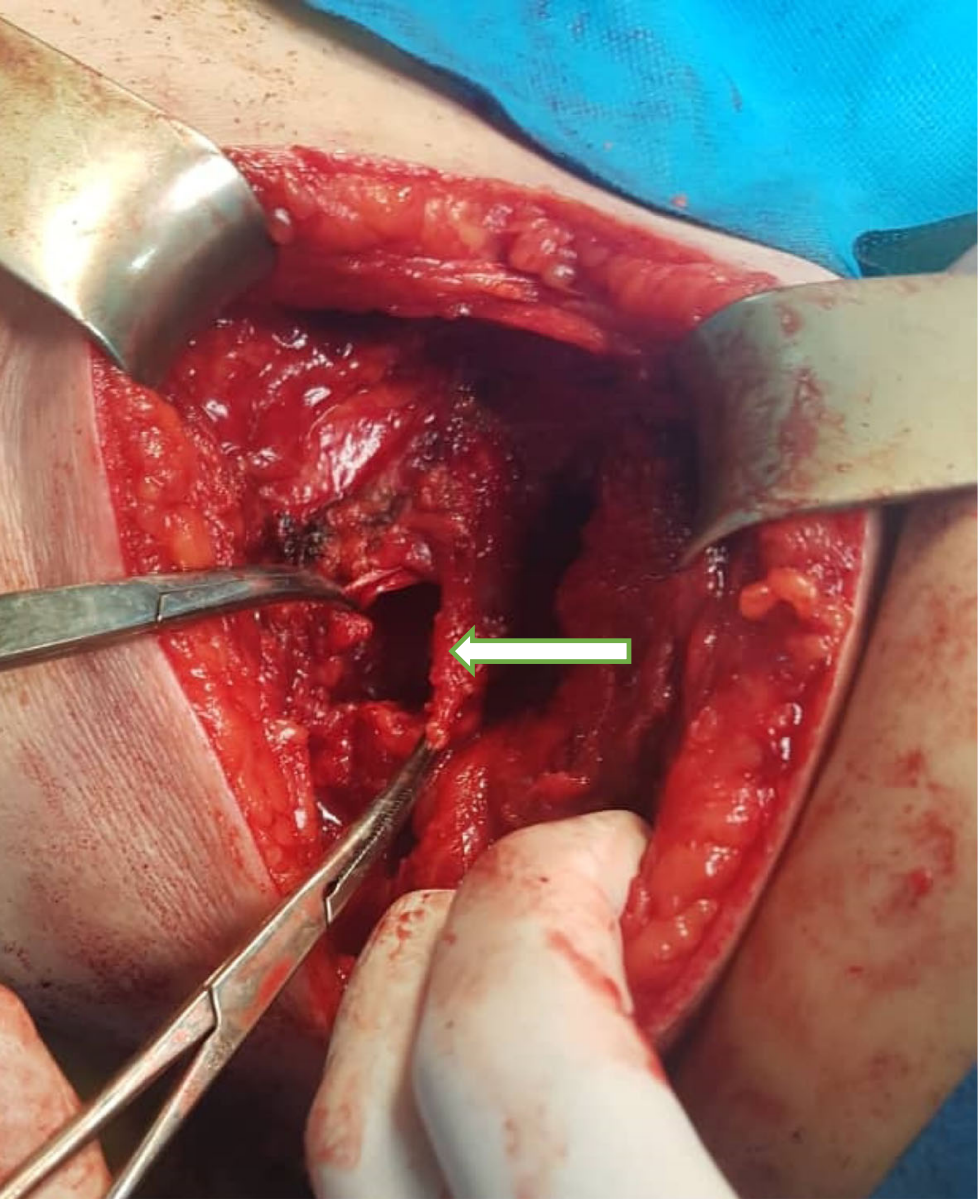
A 3 cm defect through which herniation occurred (white arrow)

**Fig. 7:**
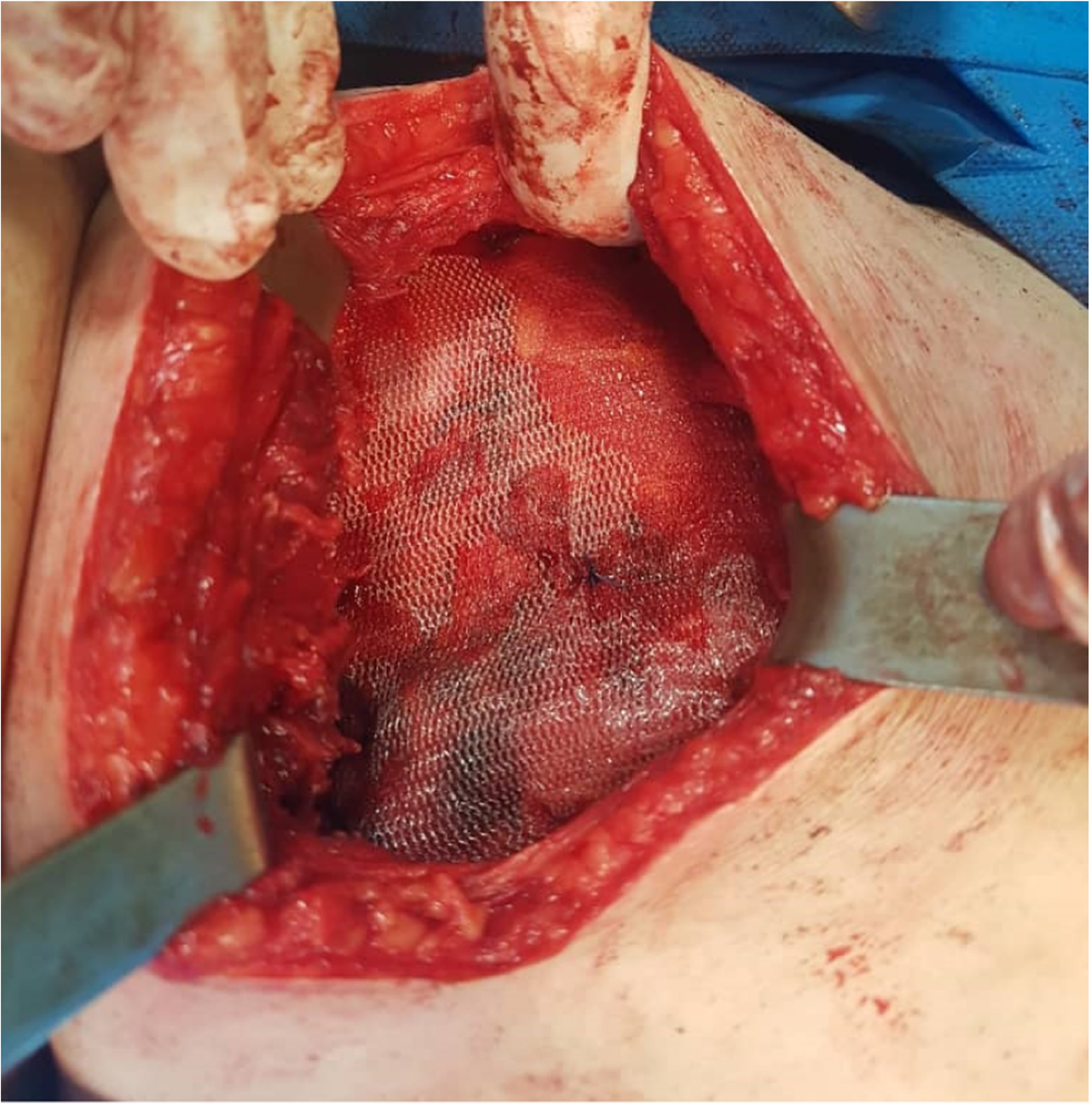
Hernioplasty with Monofilament Polypropylene Mesh

A computed tomography (CT) scan of the abdomen revealed a large herniated sac (60*30 mm) beneath the right 12th rib in the upper lumbar triangle with protrusion of retroperitoneal fat, right renal pelvis, ureteropelvic junction and proximal ureter with consecutive hydronephrosis extended to the posterior aspect of the kidney (Figs. 3 and 4). After conducting preoperational evaluations and informed consent, the patient was operated under general anesthesia in a right lateral position. A transverse incision about 10 cm was made on the bulging area. After dissection of skin, subcutaneous tissue, and muscle layer (latissimus dorsi), a hernia sac through a 3 cm defect was found. There was a herniated retroperitoneal and omental fat in the sac, so it was reduced back and closure of the abdominal wall defect was done using retro-muscular or sublay Monofilament Polypropylene Mesh (Paha® 8*15 cm) (Figs. 5, 6 and 7). Mesh was fixed to the fascia with non-absorbable interrupted sutures using a tension-free technique. The muscles and fascia were sutured separately with absorbable sutures, and the wound was closed. A drain catheter was not necessary to be placed. The operating time was 41 min, there were no hospital postoperative complications, and we discharged the patient 24 h after the surgery. The patient was visited and followed up on the 7th, 30th^,^ and 60th days after discharge from the hospital, and there were good results.

## Discussion and conclusions

Lumbar hernias are rare congenital or acquired diseases (20% vs. 80% of the cases). Congenital hernias are seen in infants and children and are related to defects in the lumbar wall. Acquired hernias are divided into two groups: spontaneous (primary) hernias and secondary acquired hernias [8]. Our patient presented a primarily acquired hernia with protrusion of retroperitoneal fat, right renal pelvis, ureteropelvic junction, and proximal ureter. Reviewing the literature regarding herniation of the urinary system into the lumbar hernia has resulted in only five previous published reports. The first report presented by Presti et al. in which a 42-year-old man had a 2-week history of intermittent right flank pain and total gross hematuria. CT scan showed a right lumbar hernia with a herniated kidney in, so then the surgical repair was done [18]. Fogarty et al. presented a 76-year-old woman with right flank pain and acute renal insufficiency. CT scan revealed herniation of retroperitoneal fat, right renal pelvis, and ureteropelvic junction through a lumbar hernia [19]. Cabello et al. presented a 79-year-old woman with a tender bulging in the left lumbar area and acute ureteral colic pain, who underwent CT scan and was diagnosed to have Grynfeltt’s bilateral hernia and herniation of left renal pelvis and proximal ureter [20]. Miyazato et al. reported a 59-year-old woman a known case of Cushing’s syndrome who underwent laparoscopic adrenalectomy and afterward developed with lumbar herniation of the left kidney [21]. Al Hooti et al. presented a 60-year-old woman with protrusion of the right kidney lower pole through the lumbar triangle 10 years after an open right pyeloplasty. She was successfully treated conservatively in clinic and received analgesics frequently for relief of mild pain without attempting operational repair [22]. The data from the previous and our current reports are summarized in Table 1.

**Table 1:** Summarized data from the previous and our current reports

Main Author	Published Year	Country	Age (year)	Sex	Chief complaint	Physical examination	Past medical History	Past surgical History	Diagnostic modality	Herniation side and incarcerated body’s part	Urological complications	Management
Presti [18]	1988	USA	42	Male	Intermittent right flank pain and total gross hematuria	a T-shaped abdominal incision directed towards the right flank, a reducible, slightly tender, solid mass in rt. lumbar area	Negative	laparotomy with partial right colectomy following a sharp injury	Abdominal x-ray, Intravenous urogram (IVU), CT scan	Right side, right kidney	right pyelocaliectasis and ureteropelvic junction obstruction	Hernioplasty
Fogarty [19]	2006	USA	76	Female	nausea, vomiting, right flank pain, and acute renal insufficiency	Not mentioned	abdominal aortic aneurysm	negative	CT scan	Right side, retroperitoneal fat, the right renal pelvis, and ureteropelvic junction	Right hydronephrosis	percutaneous nephrostomy tube, antegrade nephrostography, antegrade internal ureteral stent placement and then hernioplasty after normalization of renal function, and subsequent nephrostomy tube removal
Cabello [20]	2008	Spain	79	Female	painful swelling in the left lumbar region and acute renal colic pain	Not mentioned	hypertension, diabetes, hypercholesterolemia, hypothyroidism, hyperuricemia, hiatus hernia, deep venous thrombosis with pulmonary embolism, chronic bronchitis and morbid obesity	negative	CT scan	bilateral hernia, left side of the renal pelvis and proximal ureter	Left hydronephrosis	Non-surgical
Miyazato [21]	2011	Japan	59	Female	intermittent left back pain for 6-month	left focal back mass and tenderness	right adrenal Cushing’s syndrome and left primary aldosteronism	laparoscopic adrenalectomy	Magnetic resonance imaging (MRI) and CT scan	Left side, left kidney and	Not mentioned	Surgery
Al Hooti [22]	2014	Oman	60	Female	right-sided abdominal pain and swelling for 2-month	surgical scar without incisional hernia, non-tender oval palpableswelling, right lumbar area, positive cough sign and reducible	right pelviureteric junction obstruction	(+) open right Anderson Hynes pyeloplasty 10 years before admission	CT sacn, IVU, and Mercaptoacetyltriglycine renal scan	Right side, lower pole of the right kidney	right hydronephrosis	Non-surgical
Mehrabi ^(current report)^	2019	Iran	76	Female	back and right flank pain and a swelling in right flank since 2- month before admission	soft, mildly tender, irreducible and smooth border right lumbar mass	DM, HTN, HLP and overweight	hysterectomy, laparoscopic cholecystectomy and urinary bladder prolapse repair surgery	CT scan	Right sided, retroperitoneal fat, right renal pelvis, ureteropelvic junction and proximal ureter	right hydronephrosis	hernioplasty

Therefore, the majority of patients were female patients. The mean age of the patients was 65.33 ± 5.342 years (with a confidence level of 95%). Pain and swelling were the dominant symptoms reported in all the patients. The pain was accompanied by gross hematuria, acute renal insufficiency, and acute renal colic pain in one patient each. The right side was more involved seen in 4 cases. Physical examination was mentioned in 4 cases, of which tenderness was found in three. Past medical history was positive for abdominal aortic aneurysm in one patient. Hypertension, diabetes, and hyperlipidemia were seen in two patients. Hypothyroidism, hyperuricemia, right adrenal Cushing’s syndrome, left primary aldosteronism, right pelviureteric junction obstruction and overweight were found in one patient each retrospectively. Previous surgery history was positive in 4 cases. Laparotomy with partial right colectomy following a sharp injury, laparoscopic adrenalectomy, open right Anderson Hynes pyeloplasty and hysterectomy, laparoscopic cholecystectomy, and urinary bladder prolapse repair surgery was seen in one patient each retrospectively. CT scan was the modality used in all the cases. Intravenous urogram (IVU) was done in two patients, and an abdominal x-ray, mercaptoacetyltriglycine renal scan, and magnetic resonance imaging (MRI) were done in one patient each retrospectively. MRI was the dominant diagnostic modality used in a patient with post-laparoscopic adrenalectomy lumbar hernia. The kidney was protruded partially in all the patients. Beside, ureteropelvic junction, proximal ureter and retroperitoneal fat were seen in 2 cases each retrospectively. Urological complications were mentioned in 5 cases, of which pyelocaliectasis and hydronephrosis were seen in all the patients and ureteropelvic junction obstruction was seen in one patient.

Two patients underwent non-surgical analgesic management. Surgery was done in 4 cases, of which opened hernioplasty was done in three and was not mentioned in one. In a study done by Vagholkar et al., an open approach was implemented [23], similar to the method done in our study. They concluded that both laparoscopic and open approaches could be done, although open mesh repair is an easier, safer, and more effective method of treating this uncommon surgical illness [23]. In two previous studies, Moreno-Egea et al. evaluated laparoscopic versus open repair in lumbar hernia surgery through some prospective studies [24, 25]. They have found that results did not vary according to morbidity and recurrence rate, although it would reduce postoperative morbidity, pain, and early return to daily routine activities.

In one case, hernioplasty was done following normalization of renal function through percutaneous nephrostomy tube, antegrade nephrostography, antegrade internal ureteral stent placement, and subsequent nephrostomy tube removal.

To our best knowledge, this is one of the first reported cases and also the first literature review on acquired herniation of the renal pelvis and ureteropelvic junction through a Grynfeltt hernia with resultant hydronephrosis.

Kidney herniation through the lumbar triangle is extremely rare, and the diagnosis requires careful clinical evaluation. CT scan is the modality of choice for the assessment. Management through surgery should be done in symptomatic patients. Both laparoscopic and open approach can be done for treating this illness; however, an open approach is an easier and safer method.

## Data Availability

The datasets used and/or analysed during the current study are available from the corresponding author on reasonable request.

## Abbreviations

CT scan: Computed tomography scan
COPD: Chronic obstructive pulmonary disease
DM: Diabetes mellitus
HTN: Hypertension
HLP: Hyperlipidemia
BMI: Body mass index
IV: Intravenous
IVU: Intravenous urogram
MRI: Magnetic resonance imaging
